# Personal

**Published:** 1891-10

**Authors:** 


### Personal.

Dr. William C. Bailey, of Albion, N. Y., has recently been appointed Professor of Theory and Practice of Medicine and Clinical Medicine, and director of the bacteriological laboratory, in the Tennessee Medical College at Knoxville No better selection could have been made to fill this vacancy, and we congratulate the faculty upon securing Dr. Bailey, though Western New York will be sorry to lose this accomplished physician.

---

Dr. Julius Pohlman, who has been in Europe for the past six months, has returned and opened an office at 367 Franklin street, for the treatment of diseases of the eye and ear, to which specialties he will hereafter give attention. Hours, 9 A. M. to 1 P. M., and 4 to 5 P. M.

---

Dr. John B. Hamilton, formerly Supervising Surgeon-General United States Marine Hospital Service, has removed to Chicago. His practice is limited to surgery and office consultations. Office, Sherman House. Hours, 11 A. M. to 2 P. M.

---

Dr. A. A. Hubbell, of 212 Franklin street, Buffalo, announces that from September 1, 1891, his office hours will be from 9 A. M. to 1 P. M.

